# Sankofa: Transcending the roots of incivility in professional nursing education in South Africa

**DOI:** 10.4102/curationis.v48i1.2683

**Published:** 2025-09-18

**Authors:** Hildeguard J.-A. Vink, Maximus M. Sefotho

**Affiliations:** 1 Department of Nursing Science, School of Health Care Sciences, Sefako Makgatho Health Sciences University, Tshwane, South Africa; 2 Department of Educational Psychology, Faculty of Education, University of Johannesburg, Johannesburg, South Africa

**Keywords:** cultural framework, incivility, professional nursing education, roots, Sankofaism

## Abstract

**Background:**

Professional nursing started with Florence Nightingale, who opened a School of Nursing in the mid-1800s. Her traditions of cleanliness, caring, peacefulness, charitability, diligence, responsibility, humaneness and compassion are still relevant to modern-day nursing. Uncivilised behaviour of students and nurse educators in professional nursing education is still evident.

**Objectives:**

The objective of the study was to critically examine the roots of incivility in professional nursing education and to apply the wisdom of Sankofa to propel a renewed South African professional nursing education.

**Method:**

The study employed a qualitative research method embedded in the interpretive framework, with an exploratory descriptive design. The study was conducted at a university-based nursing school and a nursing college in the Western Cape province. 25 participants (10 nurse educators and 15 nursing students) were recruited through purposive sampling. Data collection was performed through semi-structured individual, face-to-face interviews. The participants consented and volunteered to participate in the study, and all discussions were confidential and private. Elo and Kyngäs data analysis was employed.

**Results:**

Conflicts with nursing norms, bureaucracy in nursing and professional nursing education, as well as the poor prestige of nursing and institutions of higher learning emerged as roots of incivility in professional nursing education.

**Conclusion:**

The principles of Sankofaism can be applied to professional nursing education. Students and other key stakeholders can position professional nursing education firmly in Sankofaism.

**Contribution:**

The article proposes Sankofaism as a framework to civil professional nursing education.

## Introduction

The founder of professional nursing, Florence Nightingale, established a nursing training school during the mid-1800s for the training of nurses in clinical and the theory of nursing (Egenes 2009). She fulfilled the pivotal role of nurse and care for soldiers during the Crimean War in 1854 (Hongbao, Young & Yan 2015). Her cutting-edge work formed the basis for contemporary best practices in nursing practice (Egenes 2009). In modern professional nursing education, her traditions such as cleanliness, caring, peacefulness, charitability, diligence, responsibility, humaneness and compassion are still relevant in the promotion of health and disease prevention (Bates & Memel 2021; Lee, Clark & Thompson 2013). Although many nurses try to preserve these traditions in nursing practice and education, there is resistance among students and nurse educators and, as evident from their behaviour. This behaviour is referred to as incivility, which is:

Social behaviour lacking courtesy, consideration or good manners on a scale ranging from rudeness or lack of respect for elders to vandalism and hooliganism through public drunkenness and threatening behaviour. (Yassour-Borochowitz & Desivillia 2016:414)

Historically, the word incivility was identified in the middle of the 15th century (Samman 2021). It originated as ‘incivilitatem’, a Latin word meaning rudeness or not to be civil, unfavourable and discourteous behaviour towards others (Etymology Dictionary 2020). Generally, incivility is referred to as unprofessional, insulting comments and actions, spiralling into violent behaviour that is not aligned to the nursing values and morals (Cassum 2018). Meissner (1986), an educator and nursing practitioner, asked a very controversial question ‘Nurses: Are we eating our young?’ She posed this question when she noticed how student nurses, newly qualified nurses and new employees suffered at the hands of more experienced veteran nurses. For over 40 years, the body of knowledge around what is meant by nurses eating the young has developed internationally (Gillespie et al. 2017). However, it is demoralising to notice that there is still no answer, and the interventions provided are not doing enough (Samman 2021). Even more disheartening is that nurse educators in professional nursing education openly report that they are uncivil towards students Vink and Adejumo (2015), while they are supposed to be role models and prepare the students for caring for individuals, families and communities with professionalism, humility, respect, dignity and compassion (Downing & Hasting-Tolsma 2016).

The prevalence of incivility in professional nursing education dents the image of nursing as a profession. The culture of respect and empathy for others as worthy human beings erodes with the loss of respect that was embedded in Botho and Ubuntu.

### Purpose of the study

The objective was to critically examine the roots of incivility in professional nursing education and applying the wisdom that Sankofa offers to move South African professional nursing education to a new beginning for the younger generations of nurses to come.

The research questions that guided the study were as follows:

- *What are the roots of incivility in professional nursing and nursing education?*
- *How can the wisdom of Sankofa be applied to propel South African professional nursing education past the roots of incivility to a new beginning?*

### Sankofaism as a framework to civil professional nursing education

Sankofa is an Akan philosophical tradition of the Adinkra communities of Ghana (Temple 2010) that advocates for a return to good values and principles that existed in African cultures, such as being civil towards others (Slater 2019). Sankofa provides opportunities to reconstitute cultural ways to help humanity move forward guided by cultural wisdom (Felder 2019). Sankofa provides guidance for the nursing profession to return to constructive and civil cultural practices. Nursing traditions were built on the culture of care (Chance 2015). In our understanding, care is underpinned by respect of the other person and a desire for their health and well-being. Nursing is a profession driven by a humanism for nursing those in need of care and civil treatment (McCaffrey 2019).

However, contemporary nursing traditions appear to be deviating from the ethos of nursing as the professional nursing education systems suffer severe losses of trained nurse educators (Hongbao et al. 2015). This is likely to undermine the ethics of care built on the humanism for care, therefore disregarding civil ways of dealing with others. The changing world is characterised by incivility in general. It is, therefore, imperative that the Sankofa approach of retroceding to gather good mannerisms from our traditional ways of living be revisited.

The philosophy of Sankofa represents an African perspective akin to that of Botho and Ubuntu. Quan-Baffour (2008:25) refers to the philosophy of Sankofa as African heritage. De Witte and Meyer (2012) consider the philosophy of Sankofa as ‘Sankofaism…the wisdom of our forefathers’. The driving force of Sankofa is to gaze back and draw wisdom from the past to bring it to resolve current undesirable situations. Incivility has become endemic in many societies today. This could be alluded to the loss of respect as Africans aspire to embrace foreign cultures.

On the other hand, the Sankofa bird is not stuck in the past; it keeps moving forward while looking back to take stock of the egg in its beak, which denotes the future (Quan-Baffour 2008). It provides the sense of hope that the exploration of the principles of the Sankofa philosophy can heal African nurses from the roots of incivility in nursing and professional nursing education. Therefore, the researchers explored the Sankofa framework as an African philosophy for transcending the roots of incivility in nursing and professional nursing education in South Africa. The purpose was to critically examine the roots of incivility in nursing and professional nursing education and applying the wisdom that Sankofa offers to move South African nursing and professional nursing education to a new beginning for the younger generations of nurses to come.

We propose the Sankofaist framework to civil nursing and professional nursing education that encompasses the following:

- Cultural awareness and renewal in nursing and professional nursing education (Quan-Baffour 2008).
- Spiritual recovery and traditional wisdom in nursing and professional nursing education (Slater 2019).
- Mending the fabric of our social order in nursing and professional nursing education (Ladson-Billings 1995).
- Indigenous knowledge in nursing and professional nursing education (Okwany 2016).

It is our contention that nursing and professional nursing education systems should promote cultural awareness and renewal found in diverse contexts as well as nursing ethos. This should be based on African spiritual recovery and historical wisdom to allow nursing societies to mend their fabric of social order and inculcate civility. The cornerstone should be indigenous knowledge education in the early phases of students entering the profession and the nursing education institution for training. Next we describe the aspects of the Sankofaist framework as proposed.

### Cultural awareness and renewal in nursing and professional nursing education

**FIGURE 1:**
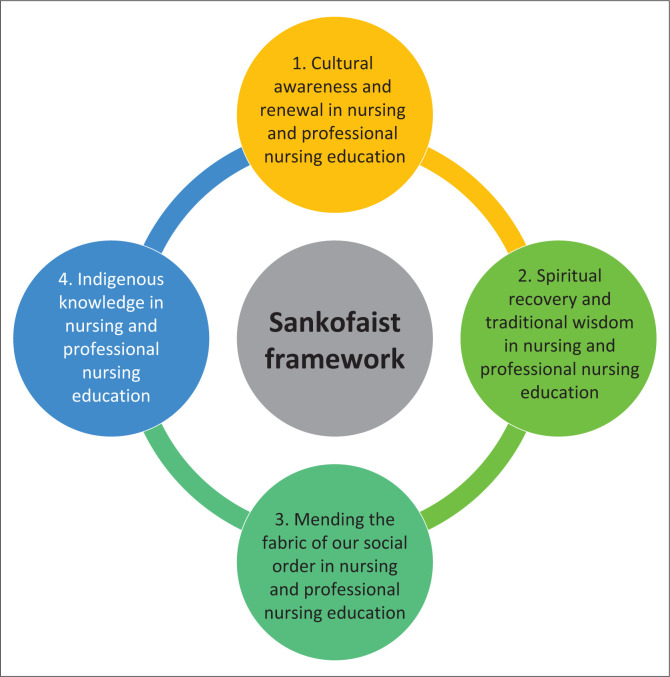
Sankofaist framework.

Cultural awareness and renewal (Quan-Baffour 2008) must be a *sine qua non* [a necessary condition] for a civil nursing and professional nursing education. Cultural awareness and renewal would be based on the Sankofa bird, which returns to its roots to gather that which was good and bring it to the future. Nursing’s attention to cultural diversity dovetails well with awareness and renewals in order to maintain civil nursing and professional nursing education (Wells 2000). Cultural awareness and renewal should be reflective of local cultures as well as the nursing culture as set out by Florance Nightingale. The Sankofaist framework as presented in Figure 1 promotes intercultural competence for the nurses of the 21st century who are confronted with caring for patients from global contexts (Moncloa et al. 2019).

### Spiritual recovery and traditional wisdom in nursing and professional nursing education

Incivility disrupts the spiritual harmony among people as it brings pain, mistrust and disrespect. Ancestral wisdom encourages spiritual harmony, and where disharmony possibly brought about by incivility reigns, spiritual recovery is recommended (Trim 2020). Spiritual recovery is perceived as cleansing and a wise move traditionally. Africans embrace cleanliness, a clean life that is based on civility and togetherness. Their cleansing rituals are performed whenever something bad happens in someone’s life. For instance, a cleansing ceremony is performed after incarceration. The Sankofaist framework encourages spiritual recovery and cleansing for individuals and communities. Where there is spiritual harmony, the fabric that makes the social order is mended in nursing and professional nursing education. The nurses’ pledge is an oath that is a sacred tradition in nursing, which allows nurses to vow their service to the public. During the induction of new nurses into the nursing profession, they undertake the oath, and on Nurses’ Day celebrations, practicing nurses renew their vows. However, the meaning of the nurses’ pledge should be upheld by nurses and nurse educators in their every encounter with patients and students to promote civility.

### Mending the fabric of our social order in nursing and professional nursing education

Today’s world is a complex one. Human relations may be broken through incivility and disharmony. When there is disharmony, social order is disturbed. Therefore, mending the fabric of our social order becomes mandatory for how well community members must interact with one another. Nursing and professional nursing education should promote harmony through civil behaviour protocol. While nursing and professional nursing education may be about healing the body and educating student nurses, healthy social relations must be promoted by eradicating incivility towards others.

### Indigenous knowledge in nursing and professional nursing education

Nursing and professional nursing education perform rituals for their nursing students from the first time they enter the profession. However, there are sometimes contributing factors that impact the socialisation process, such as socio-economic, political and cultural backgrounds, to name a few. The wealth of knowledge from indigenous knowledge systems could serve as a guide to nursing students and educators to be civil with others. Teaching and learning in professional nursing education programmes should be built on indigenous knowledge systems from as early as first year for nursing students. Nursing students are the future professional nurses and educators and need to know themselves and must be able to face the rigours and challenges of today’s ever-changing nature of nursing and professional nursing education in a professional manner and not through uncivil behaviour. Teaching early about cultures in general, as well as the nursing culture and identity, gives them pride and strategies to be resilient in the face of adversity. Indigenous knowledge also provides students with a sense of belonging.

## Research methods

### Study design

A qualitative exploratory descriptive design was selected to guide the methods and techniques for exploring and describing how Sankofa can be integrated to overcome the roots of incivility in the South African professional nursing education (Hunter, McCallum & Howes 2019). This design is inherently simple, adjustable and practical (Doyle et al. 2020). Therefore, the researcher selected it to investigate the phenomenon in its natural environment, which is a nursing education institution where students study and nurse educators are employed.

### Study population and sampling

A university-based nursing school and a nursing college collaborating with a University of Technology (UoT) in the Western Cape province, South Africa, formed the research settings. The registered undergraduate students totalled 859 with 26 academic staff members teaching undergraduate and postgraduate programmes at the university-based nursing school. At the nursing college in partnership with a UoT, there were four main campuses with a student population of 939 in the Diploma and Bachelor of Technology programmes. Fifty-four academic personnel were employed in the four campuses. The researchers were interested in the experiences of uncivil behaviour by nurse educators teaching undergraduate 4-year degree and diploma programmes, employed full-time or on permanent contractual employment. The researchers also wanted to determine insights on the opinions of undergraduate students registered in the 4-year degree and diploma programmes on their experiences of incivility in nursing schools.

A variety of nurse educators and students from different backgrounds and experiences in undergraduate nursing programmes formed part of the study population. The data collection process was initiated through purposive sampling and data saturation was reached with 23 participants. Suitable participants were purposefully sampled from the professional nursing education population to answer the research questions to address the problem of the roots of incivility in nursing education and how the wisdom of Sankofa can be applied to propel South African professional nursing education past the roots of incivility to a new beginning. Purposive sampling allows for drawing on contrasting perspectives, thereby elucidating the relevance of the position of participants against the research in question (Campbell et al. 2020). Initially from each of the two main research sites, eight participants were targeted; however, 25 participants (10 nurse educators and 15 nursing students, inclusive of both sites) demonstrated interest in participating.

### Data collection instruments and procedures

Semi-structured, individual, face-to-face interviews were conducted as the most appropriate data collection method. It allowed for interaction with the informants in obtaining their understanding of the roots of incivility in nursing and professional nursing education in the Western Cape province, South Africa, and how the philosophy of Sankofa can assist nurse educators and students to move past these obstacles. The researchers utilised open-ended questions and probes were introduced for clarity during the audio-recorded interviews. Before the interviews could take place, the participants had to give permission for the interviews to be recorded and for the researchers to make handwritten notes. Each interview lasted between 30 min and 60 min, and data saturation was obtained at 23 participants. The participants were reassured that all information relating to the interviews will be kept under lock and key, and only the researchers could access this information.

### Data analysis

Content data analysis method was used for inductive reasoning to give descriptions of the roots of incivility from the views of nursing students and educators in professional nursing education in the Western Cape province, South Africa. It was applied comprehensively as the researchers wanted to avoid fragmenting and losing the essence of the collected data. It provided a very rich and deep understanding of the data when the words could be divided into content-associated categories that describe the investigated phenomenon (Elo & Kyngäs 2008). Data analysis commenced after the completion of individual participants’ transcripts. After data saturation was reached, the researchers continued with the data analysis process for more familiarisation with the data through thorough reading of each participant’s transcript and repetitive listening to each audio recording. Thereafter, the researchers identified units of relevance, through words, phrases, paragraphs and sentences that had similarities (Elo & Kyngäs 2008; Polit & Beck 2014). The researchers continued with further organising of the data after preparing it through open coding (Polit & Beck 2014). The units of analysis were identified by the researchers and notes with headings on the side of individual participants’ transcripts were made (Burnard 1991, 1996; Hsieh & Shannon 2005, cited in Elo & Kyngäs 2008). This was followed by transferring similar units of meaning onto a spreadsheet and grouping it together under a broader category through the selection of words that described the research questions.

The researchers carefully reflected on the patterns of behaviour described and these were further explained for their meaning (Dey 1993; Kyngäs & Vanhanen 1999; Robson 1993, cited in Elo & Kyngäs 2008). Data consistency was ensured through the verification of an independent coder on the analysed data. Thick and rich descriptions of the meanings of the main category and sub-categories, where applicable, were included in the findings. These included verbatim quotations, comments and stories from extracts of the different participants’ transcripts (Streubert & Carpenter 2011).

### Ethical considerations

Ethical clearance to conduct this study was obtained from the University of the Western Cape’s Senate Research Committee (reference number 14/10/31). The researchers obtained permission from the nursing education institutions in a province of South Africa, identified as study sites, before data collection commenced for the pilot and the main study. Participation was voluntary, and informed consent was obtained after participants have been informed about the purpose of the study as well as the reasons for audio recording. No real names were used to ensure anonymity. Neuman (2014) stated that the individual researcher needs to take accountability and responsibility for ethics in research as a form of morality even if participants are not raising or demonstrating concerns and awareness with the ethical principles of the research study. The study sites and the province were omitted to ensure confidentiality. The researchers secured the participants’ interview guides and electronic information with passwords only known to them. The participants were informed that they could withdraw from the study at any given time if they experienced any discomfort during the study. The participants also had the option to be referred to counselling services if required as the information shared was sensitive.

## Results

### Theme: Roots of incivility in nursing and professional nursing education

From the data analysis, conflicts with nursing norms, bureaucracy in nursing and professional nursing education, as well as the poor prestige of nursing and institutions of higher learning emerged as roots for incivility.

#### Category 1: Conflicts with nursing norms

Professional conduct in nursing is guided by set standards and norms and participants described that students found these to be too prohibitive and old school. The influence of new developments in nursing and higher education caused students to disagree with the values of their profession. Cultural issues were also raised as contributors to how students and some nurse educators perceived nursing standards and norms. The following extracts support this view:

‘Well, they will be challenging and wear uniforms that sort of not conform to the norms and challenge that and if you do speak to them we are less … I think most of the lecturers are more open to allowing them, but we sometimes have big problems with the placement areas where they can really challenge the students.’ (Participant 7: Educator)

‘It comes from old back in the day nursing and the military used to go very closely together that is why we still like wear those shoulder bars and whatever the case may be.’ (Participant 14: Educator)

‘I can see sometimes with the students here and the hair and stuff it is not it is frowned upon, but it is their traditional way of wearing their hair and one should in the profession make provision for that and change and accept that it is not only the one culture that should be superseded.’ (Participant 7: Educator)

One student expressed that her fellow students lack insight to understand the importance of dress code as part of the professional code of conduct, and that uniformity gives nurses an identity and patients the trust that they are in good hands and will be nursed back to good health. Therefore, compliance with uniform policies is compulsory for all nurses in the clinical settings. Participant 21 stated:

‘Firstly their dress code, they do not dress like a proper nurse should dress. They come to work with hair standing wild; they got these long nails with nail polish on.’ (Participant 21: Student)

#### Category 2: Bureaucracy in nursing and professional nursing education

The younger nurse educators and some more experienced ones concurred with students that transformation in nursing is needed to embrace the current advancements. However, others opposed these sentiments and they explained that the standards of nursing need to be strengthened as they are not strong enough. Therefore, incivilities afflict the nursing teaching and learning environment. The younger nurse educator participants shared how the red tape in professional nursing education, such as rank orientation, makes junior staff members to understand the meaning of seniority and power. Their feelings of domination and oppression were compared to unprofessional behaviour in nursing education. When they explained the impact of the hierarchical system of management in professional nursing education, their emotions were very high:

‘I think it is that military you know almost military kind of subordinate behaviour that you expected to have. As academics, we need to step outside of that because we work together, we don’t work below each other, or I don’t work for a particular professor. I have a separate role and I think that the origin lies actually in how they understood the nursing profession from where they came from, and these are people that have been in nursing for 40 years you know, and they so entrenched in their behaviours that it is difficult to even address the situation.’ (Participant 14: Educator)

‘Unlike well I was not there when you trained probably. I was not there when nursing started but then according to the way I understand where nursing comes from. I mean you will find apparently if a matron walks in the ward then everyone stands and all. You don’t get that anymore now, now when a matron comes in a ward it is like there is nothing. The students they go to the ward they see those things and that is why I am saying.’ (Participant 15: Educator)

Apparently, the younger generation of nurse educators do not feel the freedom to embrace new trends in nursing and nursing education under the pressure of more experienced nurse educators that accepts change slowly. This rigidity of some participants were the root causes of incivility in professional nursing education. Nurse educators are resistant to change and cannot adopt new ideas easily. They do not want to get out of their comfort zone and they prefer to do things the way they were done years before. Participant 5 opines:

‘So, it is just from an education point of view. Many of the educators I think were older, because I think the nursing education attracted their experienced nurses. So, we brought that same experience of the top-down punitive approach from the hospital setting. Stand away matron is coming, and those older educators brought those same rules and regulations to a College or University classroom setting.’ (Participant 5: Educator)

#### Category 3: Poor prestige of nursing and institutions of higher learning

Some participants believed that nursing had lost its prestige in South Africa. The pride and dignity that nurses had for their profession had been devalued. Currently, the perceptions are that it provides job security and that students selecting nursing studies lack love and passion for the profession. Reportedly, students sometimes feel a sense of embarrassment to be nurses as the public does not have a high regard for nursing as a career and profession. Adding on this lack of prestige, some educational institutions were also regarded as a contributing factor of incivility. Therefore, students in this study experienced different types of incivility, as fellow nurses at the clinical practice settings referred to their programme as below standard and that their institution produces professionals of lower quality. However, other students and nurse educators openly admitted feeling a sense of disrespect towards their institution because of the poor working and teaching environment. According to Participants 6 and 13:

‘We have not really experienced it that much but in the clinical environment, you also get these practising professionals telling us that your 4-year curriculum is useless. You are incompetent it is almost like a repeat of what I got in the 1st year.’ (Participant 13: Student)

‘They don’t take nursing seriously and I think that they are here for the wrong reasons. They are here because of the fact that this is a place for them to stay; it is food to eat, and their motivation for coming here is wrong.’ (Participant 6: Educator)

‘In the clinical environment if I can diverge to the clinical environment there is a lot, there is a lot. The mere fact that you can be from [*sic*] just being from [*sic*] you will experience incivility from the practising professionals in the field. So, you would also be burdened with that to prove yourself that I can also be clinically competent even though I am from [*sic*].’ (Participant 13: Student)

## Discussion

The nursing profession has been described in this study to be like the military that is strict, unchangeable, outdated and not appealing to the modern-day nurse. Younger nurse educators, in particular, had disagreements with the nursing rules and thought of nursing education as bureaucratic in nature. The status of some nursing and nursing education institutions was also under question and made participants feel uncomfortable. Intimidation, lateral violence and animosity have been found to be part of the nursing profession over the years. Therefore, illuminating the roots of incivility was necessary, and spiritual harmony discourse is needed to move forward in both nursing and professional nursing education to preserve the good ethics (Szutenbach 2013).

The colour white is part of nursing culture and is symbolic of purity and cleanliness. Some participants associate the white uniform with the symbolic nursing norms, while other participants believed that nursing is far more than the white uniform. The nursing students experienced various issues in the clinical placement platforms as a result of non-compliance with the nursing dress code. The confrontations between professional nurses and students have been reported in this study. Students, although not always conforming to what the clinical settings prescribe, need role models that are consistent and correct them respectfully.

The cultural believe systems of South Africa might also have influenced the nursing norms. The participants had strong beliefs that they wished to wear hair styles and argued that because of their ethnic backgrounds, they wish to wear hair styles that resemble their materials. However, others felt it could be offensive in the clinical context as we need to be considerate of cultural diversity. The National Nurses Uniform Policy (2024) clearly outlines that hair should be neat and clean at all times. If it is long, it should be shoulder length, tied up and should fit into a theatre cap. This applies to wigs and extensions. Even brightly coloured wigs and extensions are not allowed, and nails must be kept short. The enforcement of this uniform policy even on nursing students might impact the cultural and societal way of living in nursing and professional nursing education and restore order. More emphasis on culture and indigenous knowledge systems is required to ensure that nursing students, as future professional nurses, provide culturally sensitive nursing through cultural awareness.

Supposedly, bureaucracy in nursing and professional nursing education encourages respect; however, the recent incidents of disorder, poor lines of communication, unequal division of tasks, conflicts and too much red tape observed by the students in the clinical placement settings facilitated the lack of respect, which appeared to become more apparent in the classroom and was transferred again back to the clinical settings. The younger academics reportedly strongly opposed hierarchy in professional nursing education and understood it to be the origin of incivility in the professional academic nurse training. Criticisms were raised that new academics were shaped to be submissive to older faculty that held on to the mentality to oppress, dominate and exert power inequality.

Samman (2021) confirmed that the historical military hospitals fostered power differentials, racial hierarchies, inequality and rank orientation as origins of incivility for the nursing profession. The researchers recommended to address incivility in professional nursing education programmes and that the origins of incivility need to be highlighted to students for a better understanding. Nurse educator participants in this study claimed that poor management did not facilitate individualism, and they expressed with strong emotions the need for unprofessionalism and being exploited by professors to come to an end. Vuolo (2018) asserted that educators openly abuse their positions and become aggressive and intimidating when exerting power over students, which frequently incite students to lose interest in the learning process as they become disengaged.

The students were also of the view that rigidity in South African nursing practices retard their growth as it does not empower them to compete equally with other countries. Some participants were in opposition with the hierarchical systems and wished that professional nursing education should be allowed to evolve. The teaching and learning strategies in the nursing classroom should not be top-down, but should include inclusive decision-making and embrace collaboration. Students in a UK study reportedly maintained that they were regarded as being at the bottom of the pile of respect because when doctors approach them, even if they were seated first, they would be expected to move and give the doctor the seat (Vuolo 2018).

It was stated by the participants in this study that nursing has lost its stature as they observed that students do not value the profession. Furthermore, they presumed that nursing was no more a calling but rather a financially stable option as most South African students received bursaries. There was a recommendation made by participants that the recruitment and selection criteria need to be reviewed as the character of the students was observed not to support the core values of nursing. Wisdom would need to be applied by the relevant interest groups to rebuild and repair the image of the nursing and professional nursing education. Nursing must be uplifted to the standing it previously had for communities to gain trust and respect for nurses again. Society must also play its role and cooperate with college administrators to ensure its community colleges are up to standard. Social phenomena must not infiltrate the nursing learning environment as it can damage the image of nursing (Yassour-Borochowitz & Desivillia 2016).

Claims were made by student participants from one of the study sites that they were referred to as useless by clinical staff as they only received midwifery training over a 6-month period from a worthless curriculum. Students felt very hurt and had to work extra hard to prove that they were competent because of their affiliation. These encounters gave them stress and they were not even sure about their choice of career. Their affiliation with this tarnished institution was associated with the production of incompetent, dangerous and poorly trained nursing professionals. On the other hand, some participants agreed that their institution was indeed a bad place and they could feel the lack of respect. Much as some students felt negative, others believed that socialisation through good interpersonal contact can assist them in developing a liking for the nursing profession.

### Recommendations

The researchers made the following recommendations based on the study’s findings:

Nursing education:

- Embracing African indigenous philosophies in professional nursing education, teaching and learning curricula to enhance the values of care, humility, respect and compassion.
- Nursing education institutions and clinical sites needs to co-construct the nursing curricula and have regular stakeholder engagements.

Nursing practice:

- ‘Permanent corrective measures that can offer possibilities of growth and renewal must be strengthened’ (Assié-Lumumba 2017:1).
- A deeper introspection and ongoing discourse into the cultures and spirituality in nursing is needed, and good traditional practices of nursing must be taken into the 21st century.

Nursing and nursing education policies:

- Policies and protocols in nursing and professional nursing education should include the promotion of positive practice and teaching environments to facilitate harmony.
- Consequence management must be clearly outlined for incivility that is inflicted on another person with the intention to cause harm.

### Limitations and further research

Clinical nurse educators and nurses’ opinions need to be integrated in planning for the future. An investigation into academic and workplace incivility needs to be explored. Even more necessary is to conduct research on incivility towards patients and its implications on healthcare delivery.

## Conclusion

The conflicts with nursing norms, bureaucracy in nursing and professional nursing education and the poor prestige of nursing and institutions of higher learning as the root causes of incivility are antithetical to the wisdom of Sankofa. These historical origins of incivility will not allow for civility to be learned by the new generation of nursing students and educators. However, the principles of the Sankofa philosophy can be applied in the South African professional nursing education curricula by using these roots critically, intelligently and patiently to examine how educators in nursing, students and other interested stakeholders can position professional nursing education firmly and turn their heads backwards to learn from the past experiences to improve the future of professional nursing and nursing education.

## References

[CIT0001] Assié-Lumumba, N.T., 2017, ‘The Ubuntu paradigm and comparative and international education: Epistemological challenges and opportunities in our field’, *Comparative Education Review* 61(1), 1–21. 10.1086/689922

[CIT0002] Bates, R. & Memel, J.G., 2021, ‘Florence nightingale and responsibility for healthcare in the home’, *European Journal for the History of Medicine and Health* 79(2), 1–26. 10.1163/26667711-bja10012

[CIT0003] Burnard, P., 1991, ‘A method of analysing interview transcripts in qualitative research’, *Nurse Education Today* 11, 461–466. 1775125 10.1016/0260-6917(91)90009-y

[CIT0004] Burnard, P., 1996, ‘Teaching the analysis of textual data: an experiential approach’, *Nurse Education Today* 16, 278–281. 8936234 10.1016/s0260-6917(96)80115-8

[CIT0005] Campbell, S., Greenwood, M., Prior, S., Shearer, T., Walkem, K., Young, S. et al., 2020, ‘Purposive sampling: Complex or simple? Research case examples’, *Journal of Research in Nursing* 25(8), 652–661. 10.1177/1744987120927206 34394687 PMC7932468

[CIT0006] Cassum, L.A., 2018, ‘Academic incivility in modern generation of nursing students’, *i-manager’s Journal on Nursing* 7(4), 6–9. 10.26634/jnur.7.4.13897

[CIT0007] Chance, E.A., 2015, ‘The practices of the traditional caring culture and western nursing culture in Cameroon’, Master’s thesis, The University of Bergen.

[CIT0008] De Witte, M. & Meyer, B., 2012, ‘African heritage design: Entertainment media and visual aesthetics in Ghana’, *Civilisations* 61(1), 43–64. 10.4000/civilisations.3132

[CIT0009] Dey, I., 1993, *Qualitative data analysis: A user-friendly guide for social scientists*, Routledge, London.

[CIT0010] Downing, C. & Hastings-Tolsma, M., 2016, ‘An integrative review of Albertina Sisulu and Ubuntu: Relevance to caring and nursing’, *Health SA Gesondheid* 21, 214–227. 10.1016/j.hsag.2016.04.002

[CIT0011] Doyle, L., McCabe, C., Keogh, B., Brady, A. & McCann, M., 2020, ‘An overview of the qualitative descriptive design within nursing research’, *Journal of Research in Nursing* 25(5), 443–455. 10.1177/1744987119880234 34394658 PMC7932381

[CIT0012] Egenes, K.J., 2009, ‘History of nursing’, in G. Roux & J.A. Halstead (eds.), *Issues and trends in nursing: Essential knowledge for today and tomorrow*, pp. 1–26, Jones & Bartlett, Sudbury, MA.

[CIT0013] Elo, S. & Kyngäs, H., 2008, ‘The qualitative content analysis process’, *Journal of Advanced Nursing* 62(1), 107–115. 10.1111/j.1365-2648.2007.04569.x 18352969

[CIT0014] Etymology Dictionary, 2020, *Incivility*, viewed 07 July 2025, from https://www.dictionary.com/browse/incivility.

[CIT0015] Felder, P.P., 2019, ‘The philosophical approach of Sankofa: Perspectives on historically marginalized doctoral students in the United States and South Africa’, *International Journal of Doctoral Studies* 14, 783–801. 10.28945/4463

[CIT0016] Gillespie, G.L., Grubb, P.L., Brown, K., Boesch, M.C. & Ulrich, D., 2017, ‘“Nurses eat their young”: A novel bullying educational program for student nurses’, *Journal of Nursing Education and Practice* 7(7), 11–21. 10.5430/jnep.v7n7P11 28781715 PMC5544026

[CIT0017] Hongbao, M., Young, M. & Yan, Y., 2015, ‘Nursing history literatures’, *Journal of Biomedicine and Nursing* 1(2), 51–69.

[CIT0018] Hsieh, H.-F. & Shannon, S., 2005, ‘Three approaches to qualitative content analysis’, *Qualitative Health Research* 15, 1277–1288. 16204405 10.1177/1049732305276687

[CIT0019] Hunter, D., McCallum, J. & Howes, D., 2019, ‘Defining exploratory-descriptive qualitative (EDQ) research and considering its application to healthcare’, *Journal of Nursing and Health Care* 4(1), (n.p.).

[CIT0020] Kyngäs, H. & Vanhanen, L., 1999, ‘Content analysis (Finnish)’, *Hoitotiede* 11, 3–12.

[CIT0021] Ladson-Billings, G., 1995, ‘Toward a theory of culturally relevant pedagogy’, *American Educational Research Journal* 32(3), 465–491. 10.3102/00028312032003465

[CIT0022] Lee, G., Clark, A.M. & Thompson, D.R., 2013, ‘Florence Nightingale – Never more relevant than today’, *Journal of Advanced Nursing* 69(2), 245–246. 10.1111/jan.12021 23311910

[CIT0023] McCaffrey, G., 2019, ‘A humanism for nursing’, *Nursing Inquiry* 26(2), e12281. 10.1111/nin.12281 30656789

[CIT0024] Moncloa, F., Horrillo, S.J., Espinoza, D. & Hill, R., 2019, ‘Embracing diversity and inclusion: An organizational change model to increase intercultural competence’, *The Journal of Extension* 57(6), 25. 10.34068/joe.57.06.25

[CIT0025] Meissner, J.E., 1986, ‘Nurses: are we eating our young?’, *Nursing* 16(3), 51–3. 3633461

[CIT0026] National Department of Health, 2024, *National nurses uniform policy*, pp. 1–11, National Department of Health, Pretoria.

[CIT0027] Neuman, W.L., 2014, *Pearson new international edition: Social research methods: Qualitative and quantitative approaches*, 7th edn., Pearson Education Limited, Harlow.

[CIT0028] Okwany, A., 2016, ‘Every mother dances her baby: Contextually responsive narratives of early childhood care and education in Kenya and Uganda’, *South African Journal of Childhood Education* 6(2), 1–9. 10.4102/sajce.v6i2.464

[CIT0029] Polit, D.F. & Beck, C.T., 2014, *Essentials of nursing research: Appraising evidence for nursing practice*, 8th edn., Lippincott Williams & Wilkins, Philadelphia, PA.

[CIT0030] Quan-Baffour, K.P., 2008, ‘The wisdom of our forefathers: Sankofaism and its educational lessons for today’, *Journal of Education Studies* 7(2), 22–31.

[CIT0031] Robson, C., 1993, *Real world research: A resource for social scientists and practitioner–researchers*, Blackwell Publishers, Oxford.

[CIT0032] Samman, E., 2021, ‘The origins of incivility in nursing: How reconstruction-era policies and organizations impacted social behavior within the nursing profession’, *Creative Nursing* 27(1), 66–70. 10.1891/CRNR-D-20-00085 33574176

[CIT0033] Slater, J., 2019, ‘Sankofa – The need to turn back to move forward: Addressing reconstruction challenges that face Africa and South Africa today’, *Studia Historiae Ecclesiasticae* 45(1), 1–24. 10.25159/2412-4265/4167

[CIT0034] Streubert, H.J. & Carpenter, D.R., 2011, *Qualitative research in nursing: Advancing the humanistic imperative*, 5th edn., Lippincott Williams & Wilkins, Philadelphia, PA.

[CIT0035] Szutenbach, M.P., 2013, ‘Bullying in nursing, roots, rationales, and remedies’, *Journal of Christian Nursing* 30(1), 16–23. 10.1097/CNJ.0b013e318276be28 23495431

[CIT0036] Temple, C., 2010, ‘The emergence of Sankofa practice in the United States: A modern history’, *Journal of Black Studies* 41(1), 127–150. 10.1177/0021934709332464

[CIT0037] Trim, J.N., 2020, ‘Ancestral wisdom and spiritual practices for healing: Decolonizing feminist theory and pedagogy’, Master’s dissertation, Georgia State University.

[CIT0038] Vink, H. & Adejumo, O., 2015, ‘Factors contributing to incivility in amongst nursing students at a South African nursing school’, *Curationis* 38(1), 1–6. 10.4102/curationis.v38i1.1464 PMC6091652 26841917

[CIT0039] Vuolo, J.C., 2018, ‘Student nurses’ experiences of incivility and the impact on learning and emotional wellbeing’, *Journal of Nursing Education and Practice* 8(4), 102–111. 10.5430/jnep.v8n4p102

[CIT0040] Wells, M.I., 2000, ‘Beyond cultural competence: A model for individual and institutional cultural development’, *Journal of Community Health Nursing* 17(4), 189–199. 10.1207/S15327655JCHN1704_1 11126891

[CIT0041] Yassour-Borochowitz, D. & Desivillia, H., 2016, ‘Incivility between students and faculty in an Israeli college: A description of the phenomenon’, *International Journal of Teaching and Learning in Higher Education* 28(3), 414–426.

